# Supplementary figures and images for: Epidemiologically Optimal Static Networks from Temporal Network Data
Source: PLoS Comput Biol. 2013 Jul 18;9(7):e1003142. doi: 10.1371/journal.pcbi.1003142 (PMC3715509; doi:10.1371/journal.pcbi.1003142)

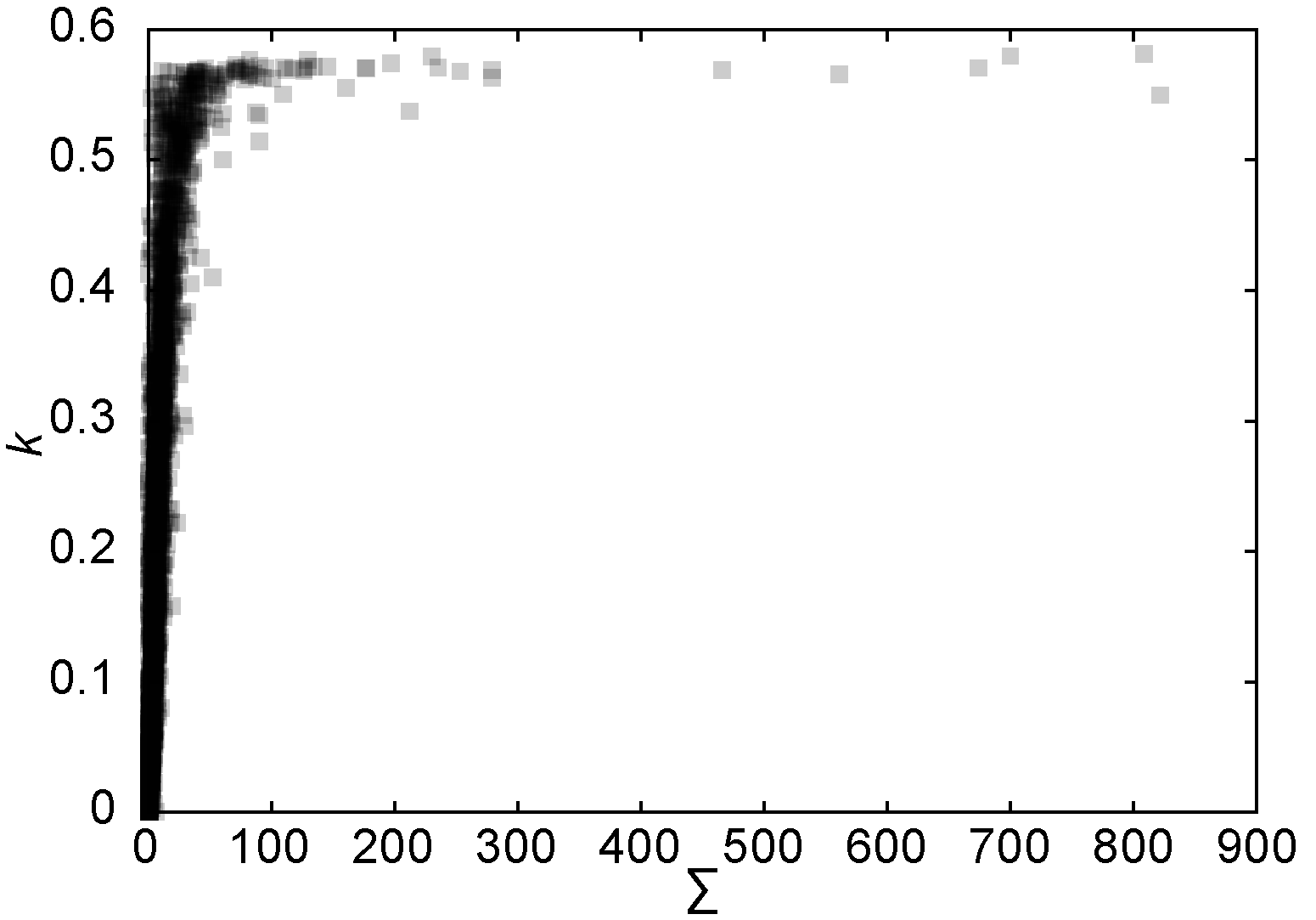

Supplement: Figure S1 — Scatter plot of the degree in a time-slice network with parameters t start = 0 and t start = T/4 as a function of the dynamic importance ∑i. The data is from the Prostitution data set. (TIF) [file pcbi.1003142.s001.tif]

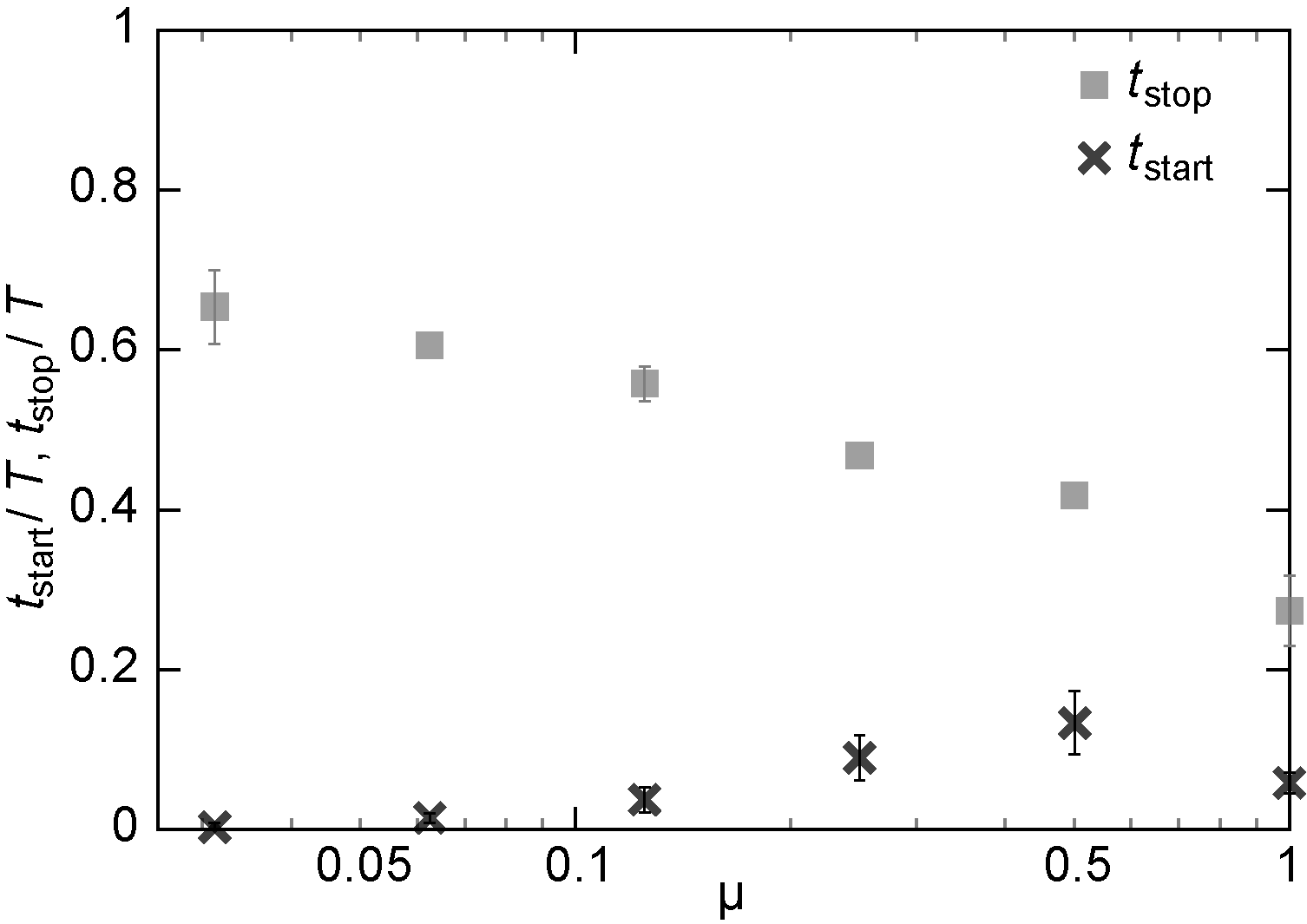

Supplement: Figure S2 — The model parameters for optimizing the time-slice networks for the synthetic data sets as a function of the overlap parameter. (TIF) [file pcbi.1003142.s002.tif]

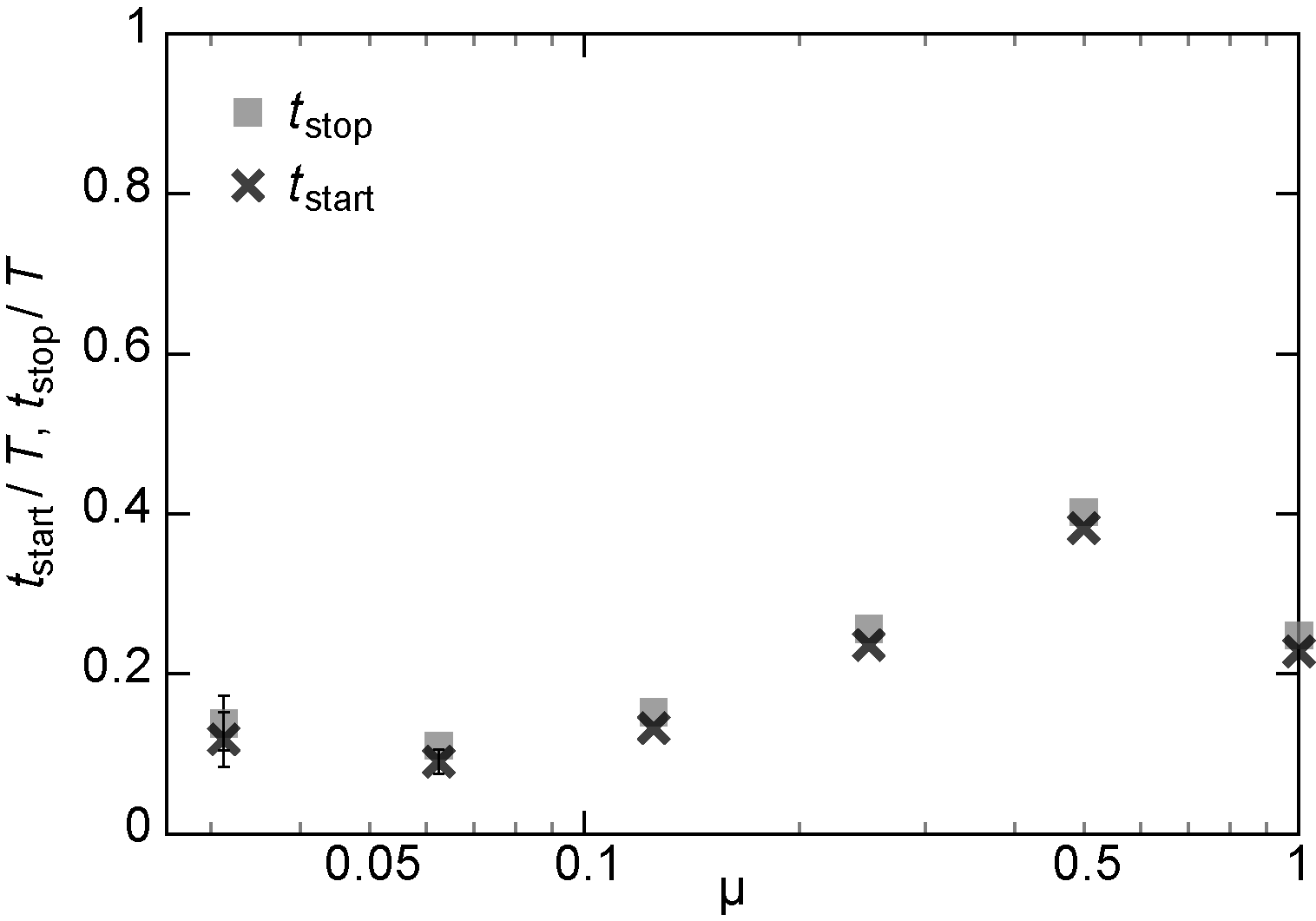

Supplement: Figure S3 — The model parameters for optimizing the ongoing networks for the synthetic data sets as a function of the overlap parameter. (TIF) [file pcbi.1003142.s003.tif]

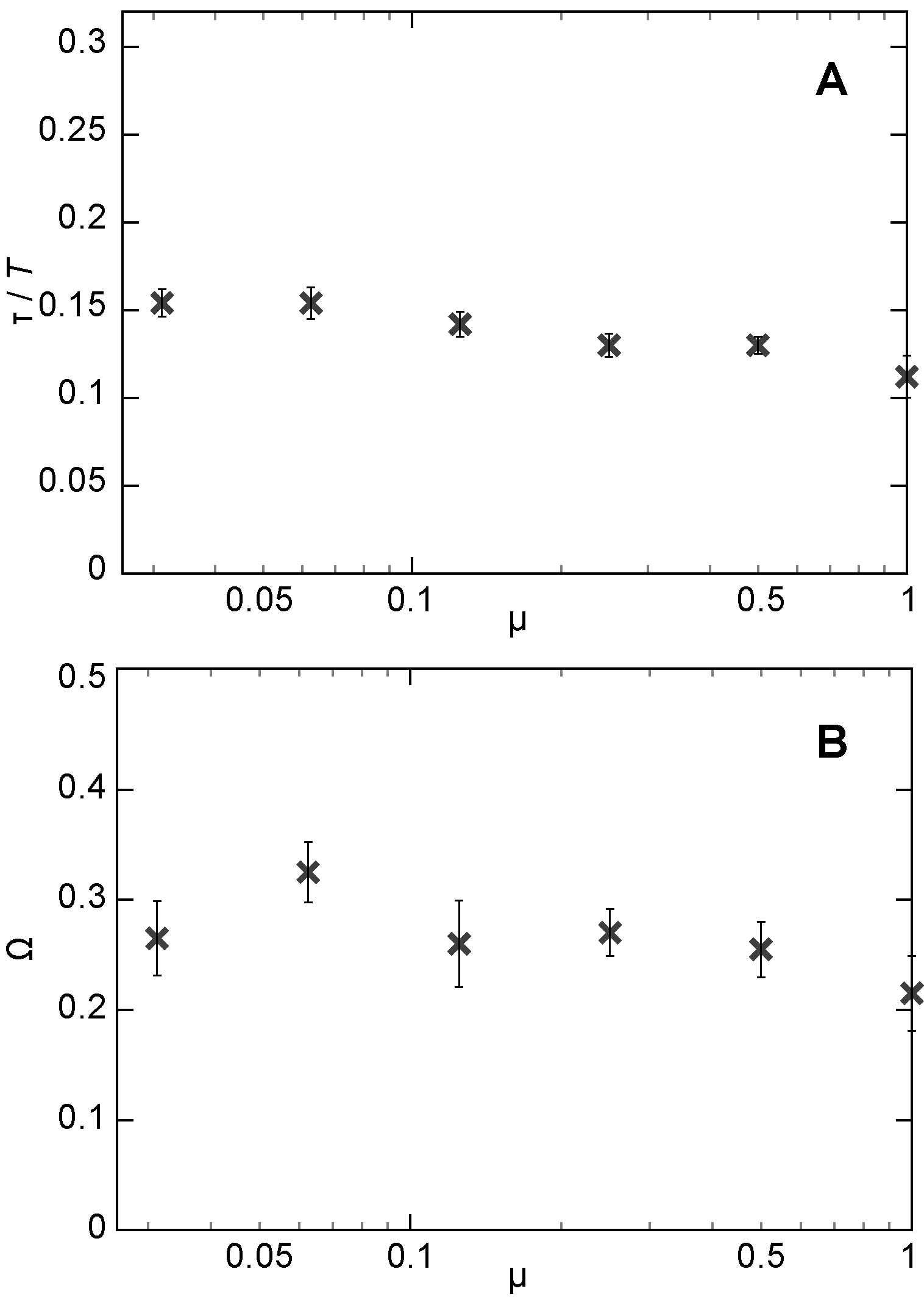

Supplement: Figure S4 — The model parameters for optimizing the exponential-threshold networks for the synthetic data sets as a function of the overlap parameter. Panel A shows values for the decay exponent τ in units of the total sampling time and B shows the τ→∞ limit cutoff Ω. (TIF) [file pcbi.1003142.s004.tif]
