# Supplementary material for: Epidemiologically Optimal Static Networks from Temporal Network Data
Source: PLoS Comput Biol. 2013 Jul 18;9(7):e1003142. doi: 10.1371/journal.pcbi.1003142 (PMC3715509; doi:10.1371/journal.pcbi.1003142)
Supplement: Table S1 — Maximal performance values for the empirical data sets. This table is exactly corresponding to Table 2 but for coreness instead of degree as an estimate for importance in static networks. (PDF) [file pcbi.1003142.s005.pdf]

Table S1. Maximal performance for the empirical data sets using coreness as importance measure for the static networks. The format is the same as Table 2 of the paper. Comparing Tables 2 and S1, we see that the results are rather similar for the degree and coreness values. In most cases, coreness outperforms degree (confirming the conclusions of Ref. [7]), but the difference is often in the third decimal of ρ_{max}. We note that the optimal performance varies quite a bit—from 0.74 for the *Prostitution* data to 0.93 for *E-mail 2*.

	Time slice			Ongoing			Exponential threshold			Acc.
	ρ_{max}	t_{start}	t_{stop}	ρ_{max}	t_{start}	t_{stop}	ρ_{max}	τ	Ω	ρ
E-mail 1	0.739(1)	0	0.42(2)	0.496(4)	0.27(2)	0.27(2)	0.775(4)	0.40(2)	0.30(2)	0.459(4)
E-mail 2	0.907(2)	0	0.25(3)	0.912(5)	0.17(3)	0.17(2)	0.930(2)	1.0(1)	0.26(1)	0.884(3)
Dating	0.829	0	0.65(3)	0.419(3)	0.25(2)	0.75(3)	0.868(2)	0.20(1)	0.20(2)	0.721(4)
Gallery	0.77(2)	0	0.72(3)	0.53(2)	0.39(3)	0.39(3)	0.87(2)	0.64(5)	0.78(3)	0.76(1)
Conference	0.778(3)	0	0.10(2)	0.759(3)	0.07(2)	0.10(1)	0.780(2)	0.60(5)	0.22(2)	0.394(6)
Prostitution	0.731(2)	0	0.77(3)	0.301(3)	0.60(3)	0.60(3)	0.742(2)	0.032(4)	0.22(2)	0.522(5)
